# Gallium-68-Labelled Indocyanine Green as a Potential Liver Reserve Imaging Agent

**DOI:** 10.1155/2019/4201353

**Published:** 2019-06-17

**Authors:** Yuxiao Xia, Li Zhang, Yanhong Zhao, Xiangdong Liu, Liang Cai, Lin Liu, Yue Chen, Wei Zhang

**Affiliations:** ^1^Department of Nuclear Medicine, Affiliated Hospital of Southwest Medical University, Luzhou, Sichuan 646000, China; ^2^Nuclear Medicine and Molecular Imaging Key Laboratory of Sichuan Province, No. 25, Taiping St, Luzhou, Sichuan 646000, China; ^3^Department of Hepatobiliary Surgery, Affiliated Hospital of Southwest Medical University, Luzhou, Sichuan 646000, China; ^4^Macau Institute for Applied Research in Medicine and Health, Macau University of Science and Technology, Taipa, Macau SAR 999056, China

## Abstract

**Objective:**

This work evaluated the potential of ^68^Ga-labelledNOTA-ICG (1,4,7-triazacyclononane-1,4,7-triacetic acid indocyanine green) for liver reserve imaging.

**Methods:**

To determine the optimal conditions for generating ^68^Ga-NOTA-ICG, various reaction parameters were implemented. Quality control analysis was performed using different chromatography techniques. The in vitro and in vivo stability was also measured at specific time points. The radioactivity ratio between n-octanol and water was determined to evaluate the water solubility of ^68^Ga-NOTA-ICG. The plasma-protein binding rate of the labelled compound was determined by the methanol method. The biodistribution and imaging findings were evaluated in normal animals at different time points after injection. A preliminary imaging evaluation was performed using an animal model of hepatic ischaemia-reperfusion injury, which was confirmed by pathology.

**Results:**

^68^Ga-NOTA-ICG was prepared with very high radiochemical purity (>98%) by reacting at 90°C for 10 min at pH = 3.5∼4.0, with excellent stability in vivo and in vitro (>95% 3 h postpreparation). The in vitro plasma-protein binding rate of ^68^Ga-NOTA-ICG was 13.01 ± 0.7%, and it showed strong water solubility (log *P*=−2.01 ± 0.04). We found that in addition to excretion through the biliary tract and intestines, ^68^Ga-NOTA-ICG can be excreted through the urinary tract. The image quality of ^68^Ga-NOTA-ICG was very high; imaging agent retained in the area of liver injury could clearly be observed.

**Conclusion:**

This is the first report on a ^68^Ga-labelled NOTA-ICG fragment for liver reserve function studies. This complex has promise as a candidate agent for liver reserve imaging.

## 1. Introduction

Liver reserve function refers to the extra compensatory potential that the liver can use when the physiological load increases. The quantitative evaluation of liver reserve function is an important basis for determining treatment options and the main reference index for evaluating prognosis. It is also a basic requirement for reducing postoperative complications and mortality. Many patients with liver diseases, especially those with hepatocellular carcinoma, often also have varying degrees of liver parenchymal damage and decreased liver reserve function [1]. The residual liver function cannot support regeneration of the liver, and the patient then develops liver failure [2]. How to accurately evaluate liver reserve function is a subject worthy of further study.

Indocyanine green (ICG) is an infrared sensitizing dye of tricarboxycyanine, which is the only dye approved by the Food and Drug Administration (FDA) for use in the body. After intravenous injection, ICG is rapidly distributed by the blood and is highly selectively taken up by hepatocytes and secreted into bile in a free form. It is excreted through the intestines, does not participate in the intestinal hepatic circulation or biotransformation, and is not excreted through other channels. The rate of ICG excretion depends on the number and function of effective hepatocytes. Based on this metabolic characteristic of ICG in vivo, ICG testing has become a widely accepted standard method for assessing liver reserve function and an important reference for clinical decision making [3]. However, the ICG clearance test results are largely dependent on hepatic blood flow. When liver disease occurs, especially liver cirrhosis, the number of effective hepatocytes is reduced, the resistance of portal vein blood flow is increased, and the blood flow of sinusoids is significantly reduced. Overall, this results in a reduction in effective hepatic blood flow and significantly influences the ICG test results. This test may also be of limited value in cholestatic patients. This dye, which is invisible to the human eye, fluoresces in the near-infrared region and thus can be detected intraoperatively (<1 cm penetration depth) using a dedicated fluorescence camera. For the last decade, ICG has been used as a tracer for sentinel lymph node biopsy (SLNB) in cases of malignancy with excellent results. Recently, a combination of ICG and radiotracers (e.g.,ICG-^99m^Tc nanocolloid) has been shown to have the advantages of both tracers [4, 5]. Positron emission computed tomography/computed tomography (PET/CT) involves imaging a radionuclide-labelled drug based on positron emission; PET/CT has higher resolution than single-photon computed tomography (SPECT/CT) and can be used in the study of tissue metabolism.

The combination of PET/CT and the ICG test is expected to achieve the bimodal fusion of optical imaging and nuclear medicine imaging such that the liver region can be accurately divided, and the functional volume of each region can be accurately calculated. To achieve this goal, a dual-modal probe based on fluorescence and nuclide imaging must first be synthesized. One of the most commonly used bifunctional connectors is 1,4,7-triazacyclononane-1,4,7-triacetic acid (NOTA), which can chelate with ^68^Ga. A substitution reaction with the carboxy terminus of ICG can be achieved at the amino terminus of the NOTA structural derivative, thereby yielding a ^68^Ga-NOTA-ICG bimodal probe. The present study was undertaken to determine the optimal labelling conditions and to evaluate the in vitro properties, biodistribution, and imaging characteristics of ^68^Ga-NOTA-ICG, which could contribute to the assessment of individualized liver function in the era of precision medicine.

## 2. Materials and Methods

### 2.1. Materials

**Figure 1:**
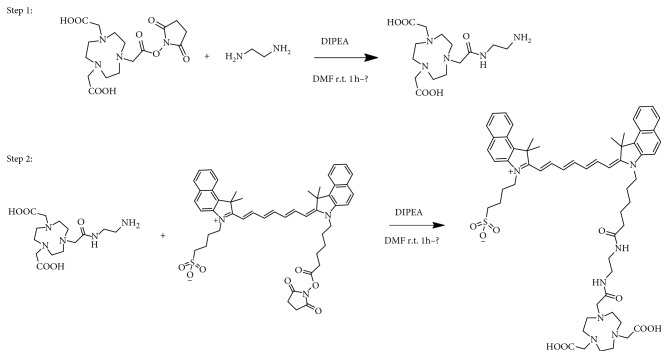
Scheme of NOTA-ICG synthesis.

^68^Ga was obtained by eluting a ^68^Ge/^68^Ga generator (740 MBq ^68^Ge/^68^Ga generator, ITG, Germany) with 0.05 M HC1. The NOTA-NHS (15 mg) and diethylamine (30 mg) were dissolved in 10 mL of N,N-dimethylformamide; then, 10 mg of N,N-diisopropylethylamine was added to catalyse the reaction, which was carried out at room temperature for 12 h. The NOTA-amino derivative was isolated and purified by high-performance liquid chromatography (HPLC). In all, 22 mg of NOTA-amino derivative and 0.5 mg of IGA-NSH were dissolved in 1 mL of N,N-dimethylformamide; then, 5 mg of N,N-diisopropylethylamine was added to catalyse the reaction, which was carried out at room temperature for 12 h. The product was purified by HPLC to yield NOTA-ICG. The synthesis of NOTA-ICG is shown in Figure 1. All other chemicals involved in the synthesis were purchased, reagent-grade materials (Aladdin Bio-Chem Technology, Shanghai, China), as were the chemicals used for assays (Kelong Chemical Reagent Factory, Chengdu, China). Paper chromatography (PC) was carried out on Xinhua No. 1 chromatography paper (Whatman-Xinhua Filter Paper Co., Hangzhou, China), and the radiochemical purity was documented using a thin-layer chromatographic (TLC) scanner (Mini-Scan; Bioscan, Inc., Washington, DC, USA) and HPLC (LabAlliance, SSI, Inc., USA). A gamma counter (SN-695B; Hesuo Rihuan Photoelectric Instrument Co., Shanghai, China) and a calibrator (CRC-15R; Capintec, Inc., Florham Park, NJ, USA) were used to measure the radioactivity of the samples. Scintigraphy was performed by PET/CT (Gemini TF/16, Philips, Netherlands) or micro-PET/CT (Siemens, Germany, Inveon MM gantry, serial number: 3125). Kunming (KM) mice, Sprague-Dawley (SD) rats and New Zealand White (NZW) rabbits were also purchased (Animal Experiment Center (animal licence SCXK 2013-17), Southwest Medical University, Luzhou, Sichuan, China). All studies were approved by the Ethics Committee of Southwest Medical University.

### 2.2. Preparation and Optimization Studies

A NOTA-ICG solution (20 *μ*g/10 *μ*L) was prepared using 30% ethanol, and ^68^Ga solution (10 mCi/mL) was prepared. We investigated the effects of NOTA-ICG dose, ^68^Ga activity, pH, temperature, and reaction time on the radiochemical purity of ^68^Ga-NOTA-ICG produced using an independent variable method. Thus, 37 MBq of ^68^GaCl_3_ solution (250 *μ*L) was added to various concentrations of NOTA-ICG solution (10 *μ*L), adjusting the respective mixtures to a pH of 3.5∼4 (using sodium acetate buffer, pH = 5.5) and allowing the reaction to occur at 90°C for 10 min. The final solution was passed through 0.22 *μ*m pore-size membrane filters.

### 2.3. Quality Control Techniques

The radiochemical yield was determined by different chromatography techniques, such as paper chromatography, TLC, and HPLC. Upon marking the origin by pencil (2 cm from one end of the Xinhua No. 1 paper), 3–5 *μ*L of the final solution was applied at the origin of the PC strips. ^68^GaCl_3_ was tested in the same manner for comparison. Finally, TLC was used to analyse the radiochemical purity. A mixture of NH_4_OAc (5 g) : 100% MeOH (50 mL) : H_2_O (50 mL) was used as the eluting solvent. The Rf value of the ^68^Ga-NOTA-ICG complex was also determined.

The ^68^Ga-labelled ICG conjugate was analysed using HPLC with a C-18 column. The elution was monitored by detecting the radioactivity signal using an NaI (Tl) detector and the UV signal at 230 nm. Water (A) and acetonitrile (B) mixed with 0.1% trifluoroacetic acid was used as the mobile phase, and gradient elution (0–2 min: 5% B, 2–25 min: 65% B) was adopted to separate the free ^68^Ga and ^68^Ga complexes. The flow rate was maintained at 1 mL/min for elution.

### 2.4. In Vitro and In Vivo Stability

To assess the in vitro stability of ^68^Ga-NOTA-ICG, the optimally labelled formulation was directly incubated at room temperature and in a water bath at constant temperature (37°C). To investigate the stability in human serum, 1.0 mL of fresh human serum was added to the labelled formulations, which were incubated at room temperature and at 37°C. All the labelled compounds were tested by TLC for radiochemical purity at 15 min, 30 min, 1 h, 2 h, 3 h, and 4 h after the reactions took place. For in vivo stability testing, four anaesthetized mice were injected intravenously with 92.5–111 MBq of ^68^Ga-NOTA-ICG in isotonic saline at a volume of 0.5 mL. Arterial plasma samples were collected at 5, 15, 30, 60, 120, and 180 min after injection. All samples were centrifuged (5 min, 3000 rpm) to separate plasma and blood cells. The supernatant was then collected and added to ice-cold methanol (methanol : plasma, 1.5 : 1). After another round of centrifugation, the supernatant was analysed by TLC for quality control.

### 2.5. Plasma-Protein Binding Rate

One millilitre of fresh anticoagulated (heparinized) human plasma was prepared. Freshly labelled compound (3.7 MBq, 0.1 mCi) was added to three centrifuge tubes, each containing 0.1 mL of human plasma. After incubation at 37°C for 2 h, 1 mL of ice-cold methanol was added to each tube. The mixtures were separated by centrifugation (5 min, 3000 rpm) to collect the supernatant, which was repeated three times. A gamma counter was then used to measure the radioactivity of the supernatant (A) and the precipitate (B) in counts per minute (CPM); the plasma-protein binding rate was calculated (PPB = B/(A + B) 100%), and the average of three tubes was recorded.

### 2.6. Lipid-Water Partition of ^68^Ga-NOTA-ICG

The lipid-water partition (P) of ^68^Ga-NOTA-ICG was determined by measuring the partition activity in n-octanol and water. In all, 490 *μ*L of ultrapure water was added to each of the three centrifuge tubes containing 0.5 mL of saturated n-octanol and combined with 10 *µ*l (1.85 MBq/0.05 mCi) of freshly prepared ^68^Ga-NOTA-ICG by ultrasonication (3 min). The centrifuge tube was allowed to stand for approximately 1 min for the liquid to become stratified. The upper and lower liquids were defined as groups A (A1, A2, A3) and B (B1, B2, B3), accounting for the organic and aqueous phases, respectively. Then, 0.1 mL was retrieved from each of the 6 sections. The radioactivity CPM of both phases was determined using the gamma counter, and the fat-water partition coefficient was calculated as follows: log *P*=log (A − background)/(B − background). The average of three tubes was recorded as log *P*.

### 2.7. Imaging and Biodistribution Studies in Normal Mice

Normal SD rats (190–240 g) were used in this study. All rats were anaesthetized by the oral and nasal inhalation of isoflurane. Immediately after each SD rat was injected with 0.5 mL of ^68^Ga-NOTA-ICG at a dose of 14.8 MBq (0.4 mCi, 50 GBq/*μ*mol), images were continuously visualized using micro-PET/CT for 3 h. After image acquisition and reconstruction were performed, the software (Inveon Research Workplace 4.2) provided by the supplier was used to delineate the blood, liver, kidney, brain, muscle, bone, stomach, lung, intestine, and gallbladder as regions of interest (ROIs) on the coronal images. The percentage of injected dose per gram of organ (% ID/g) could then be obtained by data processing.

### 2.8. Imaging Study of Rabbits with Hepatic Ischaemia-Reperfusion Injury

Normal NZW rabbits (800–950 g) were anaesthetized by an intraperitoneal injection with 5% chloral hydrate. All rabbits underwent laparotomy, and noninvasive arterial clips were used to block the blood supply to the left lateral lobe of the liver. After blocking the blood supply for 60 min, the arterial clip was opened to restore blood flow. The left outer lobe of the liver changed from dark red to bright red, indicating successful reperfusion, and then the abdominal cavity was sutured. Aseptic operations were strictly performed throughout the experiment. After surgery, the rabbits were placed in an environment at 23–26°C for 24 h. Then, the rabbits were anaesthetized again and injected with 1 mL of ^68^Ga-NOTA-ICG at a dose of 55.5 MBq (1.5 mCi, 50 GBq/*μ*mol of ^68^Ga-NOTA-ICG). The rabbits were then secured in the supine position. The rabbits were imaged by PET/CT at specific time points after the injection.

## 3. Results

### 3.1. Radiolabelling and Quality Control

**Figure 2:**
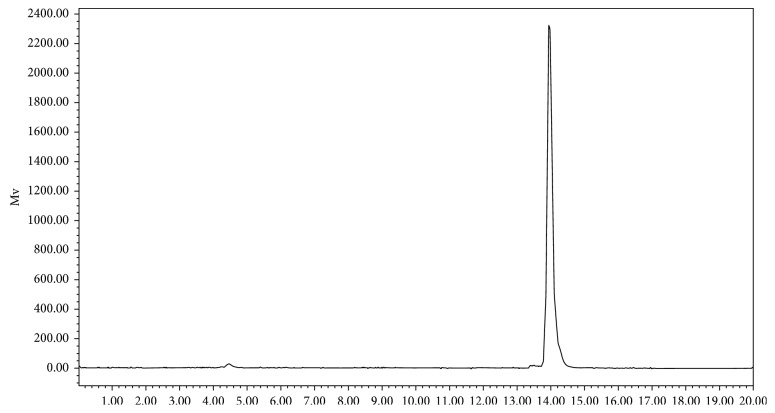
HPLC radiochromatogram of the ^68^Ga-NOTA-ICG complex showing a retention time of 13.9 ± 0.137 min (*n*=5).

Optimal radiolabelling was achieved upon the addition of 1 mL of ^68^Ga (370 MBq−555 MBq in 0.05 M HCl) to NOTA-ICG (10 *μ*g) in a kit vial, resulting in pH 3.5–4.0, and further incubation at 90°C for 10 min. ^68^Ga^3+^ (Rf = 0) remained at the point of origin, whereas the labelled compounds (Rf = 0.5–0.6) moved to the solvent front under the solvent condition (NH_4_OAc: MeOH: H_2_O). A radiochromatogram of the ^68^Ga-NOTA-ICG complex in the optimized HPLC system is shown in Figure 2. HPLC analysis of the ^68^Ga-NOTA-ICG complex showed ≥98% radiochemical purity. The retention time of ^68^Ga-NOTA-ICG in the optimized HPLC system was 13.9 ± 0.14 min, while the retention time of ^68^GaCl_3_ in the same gradient solvent system was 4.4 ± 0.06 min.

### 3.2. Characteristics of the Labelled Compounds

The stability of the labelled complexes was measured by PC at room temperature and 37°C, and the results showed that the in vitro labelling efficiency of ^68^Ga-NOTA-ICG was greater than 95% at 4 h. TLC analysis of the plasma samples demonstrated the stability of ^68^Ga-NOTA-ICG, which was 95% after 3 h, with no metabolism. The PPB rate of the labelled compound was assessed using previously described method; a rate of 13.01 ± 0.7% was determined for ^68^Ga-NOTA-ICG in vitro. To assess the water solubility of the labelled compound, the radioactivity ratio between n-octanol and water was determined. A low mean log *P* value of −2.01 ± 0.04 was calculated for ^68^Ga-NOTA-ICG, indicating strong water solubility but poor fat solubility.

### 3.3. Biodistribution

The biodistribution of ^68^Ga-NOTA-ICG in normal rats at different time points postinjection was studied in normal rats. ^68^Ga-NOTA-ICG was removed from the urinary tract, biliary tract, and intestines over time postinjection. The biodistribution of ^68^Ga-NOTA-ICG (% ID/g) in rats at specific time points is shown in Table 1.

### 3.4. ^68^Ga-NOTA-ICG Imaging

**Figure 3:**
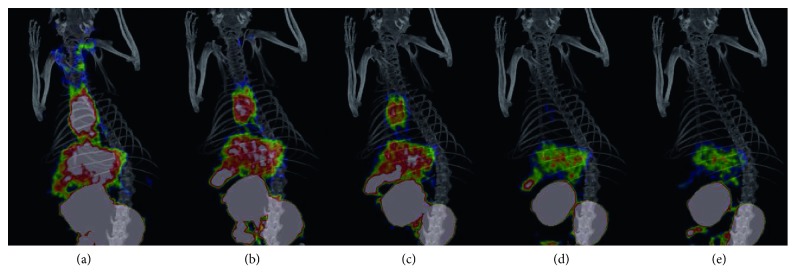
3D micro-PET/CT images of SD rats at 15 min (a), 30 min (b), 1 h (c), 2 h (d), and 3 h (e) after being injected with ^68^Ga-NOTA-ICG.

Images of ^68^Ga-NOTA-ICG in normal SD rats were obtained at various time points. At 15 min after injection, there was significant activity in the blood, liver, and kidneys (Figure 3). 3D imaging of ^68^Ga-NOTA-ICG showed strong radioactive uptake in the liver of SD rats, with excretion over time; in addition to excretion through the biliary tract and intestines, ^68^Ga-NOTA-ICG was also excreted through the urinary tract (Figure 3).

### 3.5. ^68^Ga-NOTA-ICG Imaging in Rabbits with Hepatic Ischaemia-Reperfusion Injury

**Figure 4:**
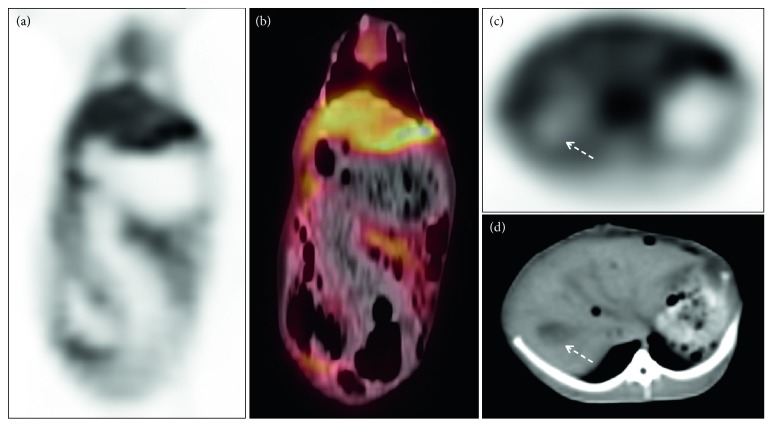
PET/CT images of rabbits with ischaemia-reperfusion injury in the left outer lobe of the liver. The coronal planes ((a) PET image; (b) PET/CT fusion image) show the retention of the radioactive imaging agent in the area of liver injury (yellow curve delineation, SUVmax = 3.4). Cross sections ((c) PET image; (d) CT image) show the gallbladder (white arrow), a small amount of gas shadow in the surgical area, and retention of the imaging agent in the left outer lobe of the liver.

**Figure 5:**
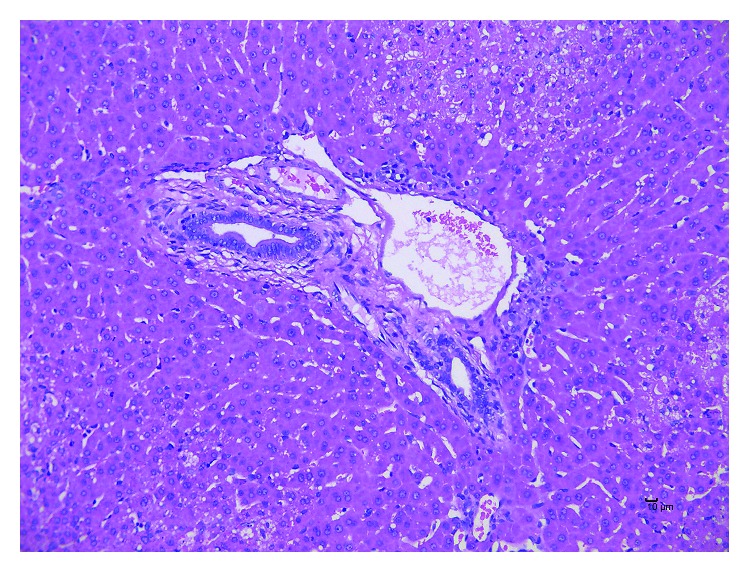
H&E staining for hepatic ischaemia-reperfusion injury. H&E staining showed hepatocytic swelling, focal necrosis, and neutrophil infiltration in the liver tissue.

In rabbits with ischaemia-reperfusion injury in the left hepatic lobe, 30 min after the injection of ^68^Ga-NOTA-ICG, the radioactivity of the left outer lobe of the rabbits was significantly increased compared with that of the other liver lobes (Figure 4). Left hepatic ischaemia-reperfusion injury was confirmed by pathology (Figure 5).

## 4. Discussion

Liver-augmenting techniques, including two-stage resection, portal vein embolization (PVE), and associating liver partition and portal vein ligation for staged hepatectomy (ALPPS), have expanded the frontiers of liver resection, resulting in an increase in the number of patients eligible for major liver resection. These aggressive surgical approaches provide the chance for curative liver resection for some patients with highly compromised livers, complex tumours, or an extensive tumour load; however, these techniques also have higher morbidity and mortality rates than standard hepatectomy. Posthepatectomy liver failure (PHLF) is the most severe complication of hepatectomy, with a mortality rate as high as 80% [6]. To avoid this complication, many studies have focused on predicting liver reserve function preoperatively. Current methods for assessing liver reserve function include serological indicators, clinical grading systems, CT volumetry, magnetic resonance imaging (MRI), dynamic quantitative liver function tests, and scintigraphic liver function tests. Serological indicators often yield inconsistent results in determining liver reserve function and have no predictive value for PHLF after hepatectomy. Among patients without cirrhosis, the Child-Pugh score shows large variations and may not reliably predict postoperative liver dysfunction. The model for end-stage liver disease (MELD) score cannot accurately predict the actual survival time of patients undergoing hepatectomy. Studies have shown that CT volumetry can guide living donor liver transplantation and hepatectomy [7, 8]. However, bile duct dilatation and vascular occlusion can cause deviations in the results of morphological techniques for estimating the inactive liver volume. Several studies have confirmed the possibility of using (Gd-EOB)-DTPA MRI for assessing liver function [9–12]. However, the molar amount of tracer required for visualization on MRI and the absolute quantification of function are technical difficulties to be solved. Dynamic quantitative liver function tests reflect global liver function, rather than the function of segments preserved after hepatectomy, and do not reflect the difference in perioperative risk caused by different resection ranges. ^99m^Tc-galactosyl serum albumin scintigraphy (^99m^Tc-GSA) is the most commonly used scintigraphic liver function test. However, imaging agent uptake measured by dynamic SPECT may underestimate liver regeneration during the early stages of the liver regeneration phase [13]. These methods each have their own limitations.

The ICG clearance test has been routinely used to assess hepatic functional reserve in many Asian centres. The results of ICG tests can be expressed in several ways [14]. In patients with hepatocellular carcinoma, normal bilirubin levels, and no ascites, the percent of ICG retained in the circulation during the first 15 min after bolus injection (ICG-R15) is a major factor in determining surgical resectability [15–17]. PET/CT has higher resolution and sensitivity than SPECT/CT, as well as a faster scanning, which is convenient for quantitative analysis with the standardized uptake value (SUV). ^68^Ga (*t*_1/2_ = 68 min; *β*^+^ : 1.9 MeV) is a nucleus that emits mainly positrons (approximately 89%), which can be used for medical diagnosis by PET/CT. It is easy to acquire and can be stably supplied by the ^68^Ge/^68^Ga generator. In addition, its half-life is relatively short, which reduces the radiation dose received by patients. A substitution reaction with the carboxy terminus of ICG can be achieved at the amino terminus of the NOTA structural derivative, thereby yielding the compound NOTA-ICG. The positron nuclide ^68^Ga labelled NOTA-ICG very quickly at a temperature of 90°C. In this study, ^68^Ga-NOTA-ICG was synthesized to effectively integrate the ICG clearance test and PET/CT, which is the most promising modality in the field of molecular nuclear medicine. Our in vitro or in vivo experiments also showed that ^68^Ga-NOTA-ICG has good stability. After ICG is injected, its blood level decreases exponentially for approximately 20 min, by which time approximately 97% of the dye has been excreted into the bile [14]. However, ^68^Ga-NOTA-ICG is partially cleared by the kidneys, which may explain why it has a low lipid-water partition coefficient. This characteristic suggests that ^68^Ga-NOTA-ICG may be less affected by biliary tract diseases, such as biliary obstruction, and is more conducive to the accurately evaluate liver function. The rapid clearance of ^68^Ga-NOTA-ICG from the body might also reduce the radiation damage to normal cells. In addition, the biodistribution results suggest little impact on the brain. In the imaging study, as expected, the image quality was so clear that the liver could be analysed even after 3 hours. In addition, we were able to quantify and visualize the increased retention of ^68^Ga-NOTA-ICG in the liver lobe affected by ischaemia-reperfusion injury. These findings indicate the utility of ^68^Ga-NOTA-ICG for assessing liver function.

The in vivo ^68^Ga-NOTA-ICG excretion pathway is different from that of ICG. The assessment of liver reserve function requires the establishment of a new evaluation system, which brings new challenges to researchers. Models of varying degrees of liver injury (e.g.,total liver injury, hemihepatic injury, hepatic lobe injury, and hepatic segmental injury) and different types of liver injury (e.g.,ischaemia-reperfusion injury, cholestatic injury, steatosis, cirrhosis, and liver tumours) need to be established to validate the efficacy of ^68^Ga-NOTA-ICG in assessing liver function. To determine this effect, a simulated surgical tangential line can be marked on the tomographic images of animal models with different degrees of liver injury. An ROI of the liver portion to be surgically removed and an ROI of the residual liver can be delineated. A mathematical function (combined with the clearance rate, SUV, and inactive liver volume ratio) can be established to quantitatively calculate postoperative residual liver function. In contrast to ^99m^Tc-GSA scintigraphy, ^68^Ga-NOTA-ICG can directly quantify residual liver function using the SUVmean, SUVmax, and other indicators and provides better image quality. The relevance of ^68^Ga-NOTA-ICG to other clinically used liver function assessment methods, such as the Child-Pugh score, the MELD score, and serological indicators, should also be studied. In addition, ^68^Ga-NOTA-ICG is also a potential fluorescent imaging agent, and intraoperative fluorescence imaging may assist liver surgeons in accurately removing nonfunctional liver tissue. The establishment of a comprehensive ^68^Ga-NOTA-ICG liver function evaluation system also requires a large number of follow-up experiments.

The authors believe that ^68^Ga-NOTA-ICG is likely to be an ideal agent for assessing liver reserve function before hepatectomy, with the following characteristics and advantages: first, accurate information on the total and local liver reserve function can be obtained. Second, the results of the assessment can be quantified and compared among patients with different diseases. Third, the liver function information obtained by this method can be processed to provide 3D images of liver function, which can directly reflect the distribution of liver function, which is helpful for simulating the resection preoperatively and assisting surgical decision making. Finally, the proposed method is minimally invasive, has excellent operability and repeatability, and will be easy to popularize. The ^68^Ga-NOTA-ICG probe is expected to allow the accurate, quick, qualitative, and quantitative evaluation of liver reserve function in patients before surgery and enable prediction of the maximum hepatectomy volume that can be tolerated to guide the modality of surgery and improve the safety of the operation.

## 5. Conclusion

We prepared ^68^Ga-NOTA-ICG with high radiochemical purity that remained stable in vitro and in vivo. The complex was quickly cleared from the body through the urinary tract, biliary tract, and intestines but could not pass through the blood-brain barrier. PET/CT imaging with this compound was performed in normal rats and rabbits with liver injury. The PET/CT images were clear and showed greater accumulation of ^68^Ga-NOTA-ICG in the area of liver injury, which could be semiquantitatively analysed. These findings suggest that ^68^Ga-NOTA-ICG has potential as a radioactive liver function imaging agent.

## Figures and Tables

**Table 1:** Biodistribution of ^68^Ga-NOTA-ICG in percentage of injected dose per gram of organ at 15 min, 30 min, 45 min, 60 min, 2 h and 3 h postinjection in normal rats (*n*=5/group).

Organ/tissue	Percentage of injected dose per gram of organ					
	15 min	30 min	45 min	60 min	2 h	3 h
Blood	0.68 ± 0.12	0.48 ± 0.16	0.32 ± 0.21	0.26 ± 0.11	0.19 ± 0.13	0.11 ± 0.07
Liver	0.54 ± 0.26	0.41 ± 0.23	0.36 ± 0.19	0.33 ± 0.13	0.27 ± 0.11	0.14 ± 0.06
Kidney	2.81 ± 1.12	1.92 ± 1.06	1.36 ± 0.47	1.24 ± 0.36	0.91 ± 0.43	0.57 ± 0.37
Intestine	0.12 ± 0.06	0.30 ± 0.16	0.34 ± 0.13	0.37 ± 0.11	1.87 ± 0.96	1.46 ± 0.83
Bone	0.16 ± 0.06	0.14 ± 0.03	0.13 ± 0.05	0.11 ± 0.03	0.14 ± 0.04	0.11 ± 0.02
Muscle	0.11 ± 0.07	0.12 ± 0.05	0.10 ± 0.04	0.12 ± 0.06	0.09 ± 0.03	0.05 ± 0.03
Lung	0.15 ± 0.08	0.10 ± 0.02	0.08 ± 0.03	0.07 ± 0.04	0.05 ± 0.02	0.03 ± 0.01
Brain	0.07 ± 0.03	0.05 ± 0.02	0.04 ± 0.02	0.05 ± 0.01	0.04 ± 0.03	0.04 ± 0.02
Stomach	0.08 ± 0.04	0.06 ± 0.03	0.09 ± 0.03	0.09 ± 0.04	0.07 ± 0.03	0.06 ± 0.02
Gallbladder	0.27 ± 0.16	0.48 ± 0.19	0.42 ± 0.18	0.31 ± 0.13	0.34 ± 0.12	0.33 ± 0.13
Liver/blood	0.79	0.85	1.13	1.27	1.42	1.27

Values are the mean ± standard deviation (*n*=5/group).

## Data Availability

The data used to support the findings of this study are available from the corresponding author upon request.
